# Responsiveness of the Indonesian Versions of the Anterior Cruciate Ligament-Return to Sport After Injury Score, the International Knee Documentation Committee Subjective Knee Form, and the Lysholm Score in Patients With ACL Injury

**DOI:** 10.1177/23259671231191827

**Published:** 2023-08-18

**Authors:** Romy Deviandri, Hugo C. van der Veen, Andri MT Lubis, Maarten J. Postma, Inge van den Akker-Scheek

**Affiliations:** † Department of Orthopedics, University of Groningen, University Medical Center Groningen, Groningen, the Netherlands.; ‡ Department of Physiology-Faculty of Medicine, Universitas Riau, Pekanbaru, Indonesia.; § Division of Orthopedics-Sports Injury, Arifin Achmad Hospital, Pekanbaru, Indonesia.; ∥ Department of Orthopedics-Faculty of Medicine, Universitas Indonesia/Cipto Mangunkusumo Hospital, Jakarta, Indonesia.; ¶ Department of Health Sciences, University of Groningen, University Medical Center Groningen, the Netherlands.; ＃ Department of Economics, Econometrics and Finance, University of Groningen, Faculty of Economics and Business, Groningen, the Netherlands.; ** Department of Pharmacology and Therapy, Universitas Airlangga, Surabaya, Indonesia.; †† Center of Excellence in Higher Education for Pharmaceutical Care Innovation, Universitas Padjadjaran, Bandung, Indonesia.; *Investigation performed at the Department of Orthopedics, University of Groningen, University Medical Center Groningen, Groningen, the Netherlands*

**Keywords:** ACL tear, ACL-RSI, IKDC, MIC, PROMs

## Abstract

**Background:**

The Indonesian versions of the Anterior Cruciate Ligament-Return to Sport after Injury (ACL-RSI), International Knee Documentation Committee subjective knee form (IKDC), and the Lysholm scores are considered valid and reliable for Indonesian-speaking patients with anterior cruciate ligament (ACL) injury.

**Purpose/Hypothesis:**

The purpose of this study was to determine the responsiveness of the ACL-RSI, IKDC, and Lysholm scores in an Indonesian-speaking population with ACL injury. It was hypothesized that they would have good responsiveness.

**Study Design:**

Cohort study (diagnosis); Level of evidence, 2.

**Methods:**

Between March 1, 2021, and February 28, 2022, patients with an ACL injury at a single hospital in Indonesia were asked to complete the ACL-RSI, IKDC, and Lysholm scores before either reconstruction surgery or nonoperative treatment. At 6 months after treatment, the patients completed all 3 scores a second time, plus a global rating of change question. The distribution-based and the anchor-based methods were used to study responsiveness. For each scale, the standardized response mean, minimal clinically important difference (MCID), and minimal detectable change (MDC; at the group [MDC_gr_] and individual [MDC_ind_] levels) for each scale were determined.

**Results:**

Of 80 eligible patients, 75 (93.8%) completed the study. The standardized response means for the ACL-RSI, IKDC, and Lysholm scores were 1.59, 1.72, and 1.51, respectively, indicating good responsiveness. The MCIDs for the ACL-RSI, IKDC, and Lysholm scores were 6.8, 7.8, and 4.8, respectively; all MCIDs were larger than that of the MDC_gr_ (1.1, 0.7, and 0.6, respectively). At the individual level, the MCID for the IKDC was larger than the MDC_ind_ (7.8 vs 5.8). However, the MCIDs for ACL-RSI and Lysholm scores were smaller than those of the MDC_ind_ (6.8 vs 10.9 and 4.8 vs 5.1, respectively).

**Conclusion:**

The Indonesian ACL-RSI, IKDC, and Lysholm scores indicated good responsiveness and can be used in the follow-up of patients after ACL injury, especially at the group level. In individual patients, IKDC was found to be more efficient than the ACL-RSI or Lysholm scores for detecting clinically important changes over time after ACL treatment.

The incidence of anterior cruciate ligament (ACL) injury has increased over the past decade. The overall age- and sex-adjusted annual incidence of ACL injuries is 74.6 per 100,000 person-years.^13^ In Indonesia, the number of ACL reconstructions rose by 42% in 2019 compared with 2018, based on number of implants (1575 implants in 2018 vs 2236 in 2019).^5^ For most patients, the primary goal of treatment after ACL injury is to return to sport, preferably at the preinjury level.^7^ This goal could be reached by either reconstruction surgery or nonoperative treatment in selected patients.^7^ To improve management of ACL injury, it is essential not only to assess outcome after treatment but also to monitor patients over time during treatment.^15^

To assess the outcome in patients after ACL injury, several patient-reported outcome measures (PROMs) are available. In 1982, the first version of the Lysholm knee score was introduced to the medical community; a modified version was introduced in 1985.^16^ The International Knee Documentation Committee subjective knee form (IKDC) was developed in 1987 to evaluate the outcome of patients with knee injuries. A revised version of IKDC was published in 2001.^9^ In 2008, Webster et al^20^ developed and studied the validity of the Anterior Cruciate Ligament-Return to Sport after Injury (ACL-RSI) score to determine psychological factors affecting return to sport after ACL treatment. All those questionnaires have been translated into Indonesian and can be considered valid and reliable for an Indonesian-speaking population with ACL injury.^4–6^ However, the responsiveness of those scores has not yet been studied.

Researchers and clinicians need high-quality PROMs; of all measurement properties, responsiveness is especially important if patients are followed over time. Responsiveness is considered longitudinal validity (ie, the degree to which an instrument is able to measure change in the measured construct).^11,17^ In longitudinal research as well as clinical practice, PROMs are used for example to determine the effectiveness of a treatment or the progress of a patient over time. A PROM with good responsiveness is able to measure change, and also the right amount of change, over time in a valid way.

This study aimed to determine the responsiveness of the Indonesian versions of ACL-RSI, IKDC, and Lysholm scores in patients after ACL treatment. It was hypothesized that the Indonesian ACL-RSI, IKDC, and Lysholm scores have good responsiveness.

## Methods

### Patients and Procedures

The study protocol received approval by the local institutional review board, and all participating patients provided informed consent. All patients with an ACL injury at a single hospital who underwent either reconstruction surgery or nonoperative treatment between March 1, 2021, and February 28, 2022, were asked to participate in this study. Patients with a revision ACL reconstruction or multiligamentous injury were excluded, as were those unable to understand Indonesian. Patients with a level of activity <5 according to the Tegner scale were also excluded, as this study relates to return to sport after injury.

All patients were asked to complete 3 PROMs - the Indonesian versions of ACL-RSI, IKDC, and Lysholm scores - at 2 different time points: before treatment (t_1_) and 6 months after treatment (t_2_). At t_1_, demographic information was provided, and current level of work and sports participation were measured with the Tegner activity scale. At t_2_, patients also completed 2 questions using a global rating of change (GRC) scale. The GRC was used to determine whether confidence in sports resumption changed between t_1_ and t_2_ (question 1) and whether health status and knee function changed between t_1_ and t_2_ (question 2). The 5 possible responses were “much increased,” “slightly increased,” “same condition,” “slightly decreased,” and “significantly decreased.” Patients who did not come to the hospital after a 6-month interval were reminded once by telephone and were excluded if they did not show up more than 1 month apart.

Patients were dichotomized into 2 categories based on their answer to the GRC questions: those who responded with “much increased” and “slightly increased” were considered as having improved, and those who reported “same condition” were considered unchanged. Patients who reported “slightly decreased” and “significantly decreased” on question 1 were left out of the responsiveness analysis for the ACL-RSI, and patients who reported “slightly decreased” and “significantly decreased” on question 2 were left out of the responsiveness analysis for the IKDC and Lysholm scores.

### Measurement Instruments

The ACL-RSI measures athletes’ emotions, confidence, and risk appraisal when returning to sport after ACL injury treatment. The score comprises 12 items, organized into 3 subgroups of psychological factors (emotions, confidence in performance, and risk appraisal). The form is scored by summing the scores for the individual items and then transforming the score to a scale ranging from 0 to 100, with 100 indicating absence of symptoms and higher levels of scores. We used the valid and reliable Indonesian version of ACL-RSI.^6^

The IKDC measures symptoms, function, and sports activity in patients with various knee conditions and has 18 questions. The scale is scored by summing the scores for the individual items and then transforming the score to a scale ranging from 0 to 100, with 100 indicating absence of symptoms and higher levels of functioning.^8^ The valid and reliable Indonesian version of IKDC was used.^5^

The Lysholm is a knee-specific scale. It includes 8 questions (limping, locking, pain, stair-climbing, support use, instability, swelling, squatting) and measures perceived symptoms and function in patients with various knee conditions. The scale is scored by summing the scores for the individual items and then transforming the score to a scale ranging from 0 to 100, with 100 indicating absence of symptoms and higher levels of functioning.^16^ The valid and reliable Indonesian version of the Lysholm scale was used.^4^

The Tegner scale assesses activity levels based on work and sports activities. It consists of a numerical range from 0 to 10; each value indicates the ability to perform specific activities, with the highest value indicating the best score. The Tegner scale was developed as an addition to the Lysholm scale.^16^ The valid and reliable Indonesian version of the Tegner scale was used.^4^

### Statistical Analysis

Characteristics of the study population and PROM scores were reported as means and standard deviations or as frequencies and percentages. Responsiveness was determined using both the distribution-based and the logistic regression-based methods proposed by Terluin et al.^17^ The distribution-based method was used to compare the results of the 2 time-interval measurements of the scales (t_1_ and t_2_) and to examine whether there was a statistically significant change in scores between the 2 measurements. The standardized response mean (SRM) was calculated; this is the ratio of the mean difference between baseline and follow-up assessment to the standard deviation of the mean.^12^ SRM values <0.5 indicate low responsiveness; 0.5 to 0.8, moderate; and >0.8, good responsiveness.^2,3^ Standard error of measurement and minimal detectable change (MDC) were retrieved from previous validity studies.^4–6^

The dichotomized GRC score was the external criterion for confidence in both sports resumption and knee function to evaluate whether the change in score was perceived as important by the patient and to determine the minimal clinically important difference (MCID). Univariate logistic regression analyses were performed for all 3 PROMs evaluated. The predictor variable was the change in scores between the 2 measurement times. MCID values with corresponding 95% confidence intervals were calculated. Diagnostic performance of the GRC was also evaluated by calculating sensitivity, specificity, positive predictive value, negative predictive value, and percentage of misclassification.

Statistical analyses were performed using SPSS Statistics Version 26.0 (IBM), with level of significance set at 5%.

## Results

### Patient Characteristics

Of 100 patients with ACL injury evaluated during the inclusion period, 20 were excluded for various reasons (revision ACL reconstruction or multiligamentous injury, n = 11; Tegner level <5, n = 9). Of the 80 patients eligible to participate in the study, 75 (93.8%) filled out and returned 2 complete sets of questionnaires. Most patients were nonprofessional male athletes, and the mean age was 27.6 ± 8.9 years. Patient characteristics are presented in Table 1.

**Table 1:** Patient Characteristics (N = 75)*^a^*

Characteristic	Value
Age, y	27.6 ± 8.9
Sex	
Male	69 (92)
Female	6 (8)
Affected side	
Right	45 (60)
Left	29 (38.7)
Both	1 (1.3)
Athletic level	
Professional athlete	8 (10.7)
Nonprofessional athlete	67 (89.3)
Education	
Middle school	1 (1.4)
High school	22 (29.3)
Bachelor’s degree	49 (65.3)
Master’s degree	3 (4.0)
Activity when injured	
ADL	4 (5.3)
Sport	68 (90.7)
Work	3 (4.0)
Tegner level	6.4 ± 1.2
Treatment	
Surgery	45 (60)
Nonoperative	30 (40)

*^a^* Data are presented as mean ± SD or n (%). ADL, activities of daily living.

### Outcomes

The mean scores of the first and second assessments for all 3 PROMs can be found in Table 2. Based on the GRC scores, 66 patients (88%) had much or slightly increased confidence, 7 patients (9.3%) reported no change, and 2 patients (2.7%) had slightly or significantly decreased confidence in sports resumption. In addition, 73 patients (97.3%) reported better knee function, 1 patient (1.3%) reported no change, and 1 patient (1.3%) reported worse function. The SRMs for ACL-RSI, IKDC, and Lysholm scores were 1.59, 1.72, and 1.51, respectively (Table 2).

**Table 2:** Mean Score on First and Second Assessment; SRM, SEM, and MDC Values of the ACL-RSI, IKDC, and Lysholm Scores*^a^*

Measure	Score, t_1_	Score, t_2_	Δ(t_2_-t_1_)	SRM	SEM	MDC_ind_	MDC_gr_
ACL-RSI	43.5 ± 16.6	69.9 ± 16.6	26.4 ± 16.6	1.59	3.9	10.9	1.1
IKDC	47.1 ± 14.2	68.6 ± 11.3	21.5 ± 12.5	1.72	2.1	5.8	0.7
Lysholm	53.6 ± 14.7	76.9 ± 12.6	23.3 ± 15.4	1.51	1.8	5.1	0.6

*^a^* Score t1, score t2, and t2-t1, data are reported as mean ± SD. ACL-RSI, Anterior Cruciate Ligament Return to Sport after Injury scale; IKDC, International Knee Documentation Committee subjective knee form; MDC_gr_, minimal detectable change for groups; MDC_ind_, minimal detectable change for individuals; SEM, standard error of measurement; SRM, standardized response mean; t_1_, first administration; t_2_, second administration.

### Minimal Clinically Important Difference

Table 3 shows the MCID for the 3 PROMs. The mean change in ACL-RSI score in the subgroup of patients who reported having more confidence in the GRC after the second administration was 29.1 ± 15.1. The mean change in the subgroup of patients who reported no change was 1.1 ± 3.8. The MCID was 6.8, with a misclassification rate of 3%.

Regarding the IKDC, the mean change in scores in the subgroup of patients with better knee function in the GRC after the second administration was 22.4 ± 11.8. The mean change in the subgroup of patients who reported no change was 1.7 ± 2.9. The MCID was 7.8, with a misclassification rate of 4%.

The mean change in Lysholm in the subgroup of patients who reported having better knee function in the GRC after the second administration was 24.4 ± 14.7. The mean change in the subgroup of patients who reported no change was 3 ± 2. MCID was 4.8, with a misclassification rate of 3%.

**Table 3:** MCID for the ACL-RSI, IKDC, and Lysholm*^a^*

Measure	MCID (95% CI)	Sensitivity, %	Specificity, %	PPV, %	NPV, %	MIS, %
ACL-RSI	6.8 (0.7-14.4)	97	100	100	78	3
IKDC	7.8 (1.1 -1.9)	96	100	100	50	4
Lysholm	4.8 (0.9-3.4)	97	100	100	60	3

*^a^* ACL-RSI, Anterior Cruciate Ligament-Return to Sport after Injury scale; IKDC, International Knee Documentation Committee subjective knee form; MCID, minimal clinically important difference; MIS, misclassification; NPV, negative predictive value; PPV, positive predictive value.

### Relationship Between MCID, MDC, and Confidence in Sports Resumption or Knee Function

**Figure 1.**
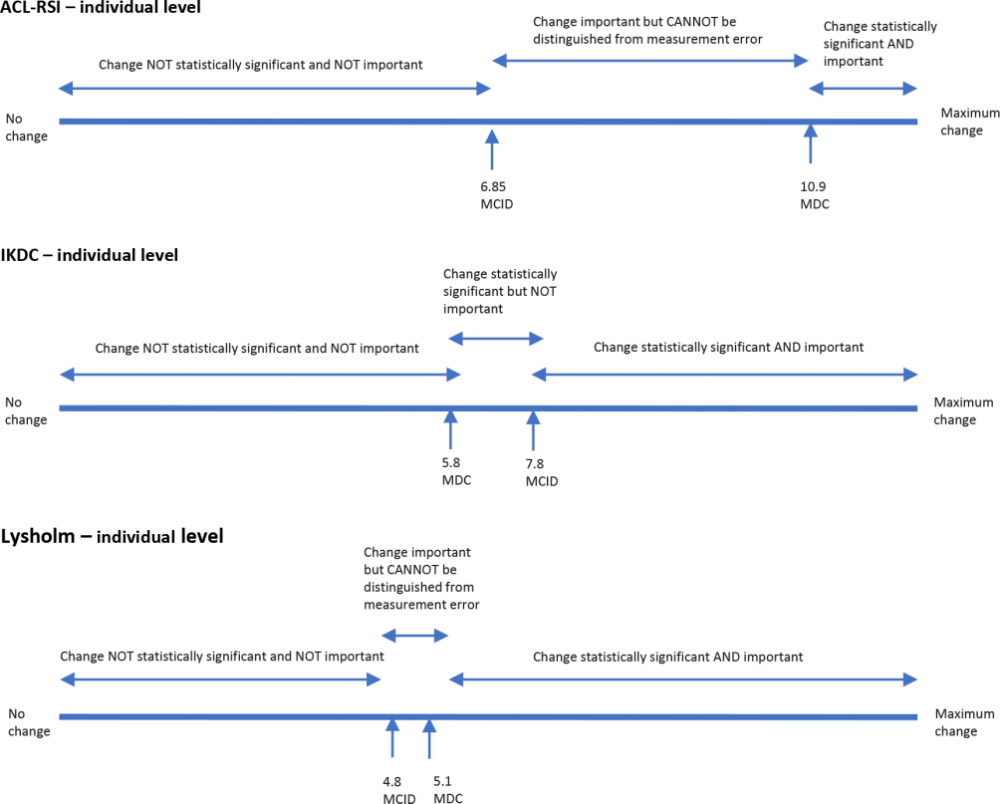
Visual relationship between the MCID and the MDC of the ACL-RSI, IKDC, and Lysholm scores at the individual level. ACL-RSI, Anterior Cruciate Ligament-Return to Sport after Injury scale; IKDC, International Knee Documentation Committee subjective knee form; MCID, minimal clinically important difference; MDC, minimal detectable change.

**Figure 2.**
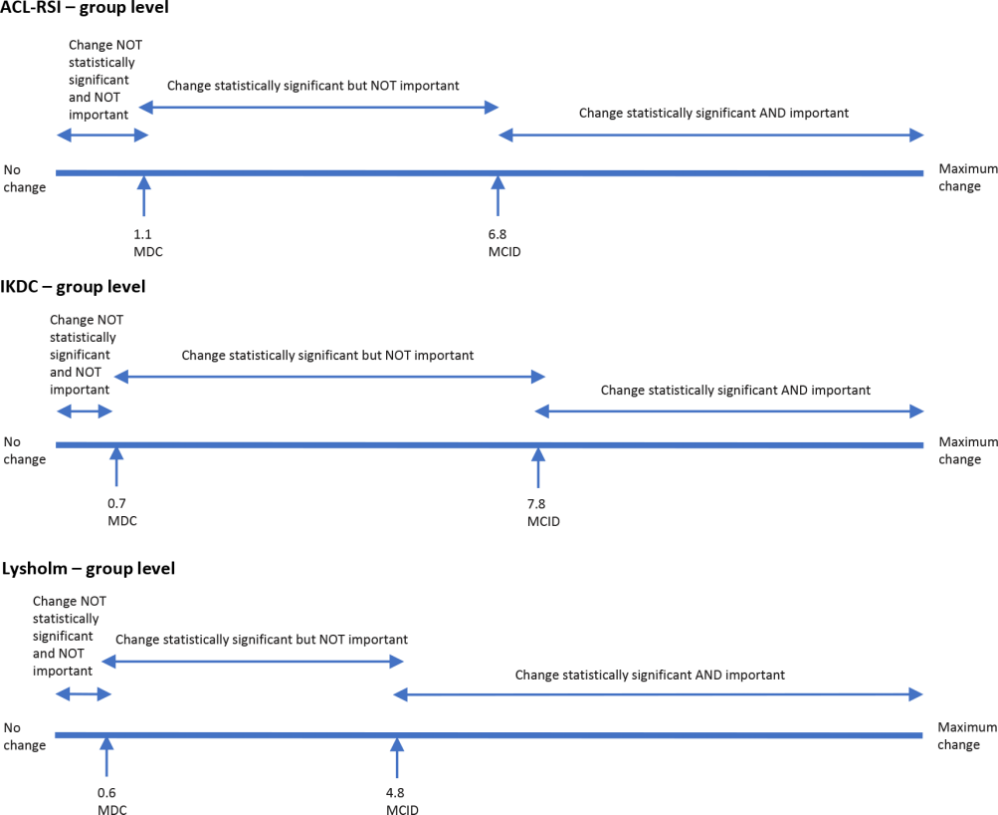
Visual relationship between the MCID and the MDC of the ACL-RSI, IKDC, and Lysholm scores at the group level. ACL-RSI, Anterior Cruciate Ligament-Return to Sport after Injury scale; IKDC, International Knee Documentation Committee subjective knee form; MCID, minimal clinically important difference; MDC, minimal detectable change.

The relationship between confidence in sports resumption and the MCID for ACL-RSI and that between knee function and the MCID for both IKDC and Lysholm are shown in Table 4. The relationship between MCID and MDC values in ACL-RSI, IKDC, and Lysholm in individuals and at the group level is shown in Figures 1 and 2.

**Table 4:** Relationship between Confidence in Sports Resumption/Better Knee Function and the MCID for ACL-RSI, IKDC, and Lysholm*^a^*

Δ(t_2_-t_1_)	GRC Scores		
	More Confidence/ Better Knee Function	No Change	Total
ACL-RSI			
≥6.8	64	0	64
<6.8	2	7	9
IKDC			
≥7.8	68	0	68
<7.8	3	3	6
Lysholm			
≥4.8	69	0	69
<4.8	2	3	5

*^a^* ACL-RSI, Anterior Cruciate Ligament-Return to Sport after Injury scale; GRC, global rating of change; IKDC, International Knee Documentation Committee subjective knee form; MCID, minimal clinically important difference.

## Discussion

The most important finding of this study is that the Indonesian versions of the ACL-RSI, IKDC, and Lysholm scores all showed good responsiveness and can be used in the follow-up of patients after ACL treatment. However, the results of the study also showed that ACL-RSI and Lysholm scores might be more suitable for detecting clinically important changes on a group level over time than on an individual level, while IKDC seems more efficient than the other 2 for both group level and individual level.

ACL-RSI, IKDC, and Lysholm scores yielded large SRM values of 1.59, 1.72, and 1.51 and MCID values of 6.8, 7.8, and 4.8, respectively. MDC at the individual level (MDC_ind_) for ACL-RSI and Lysholm scores (10.9 and 5.1, respectively) were larger than the respective MCID values. MDC_ind_ for IKDC was smaller than the MCID (5.8 vs 7.8). From these results it can be concluded that, to be clinically relevant as well as statistically significant when monitoring changes in individual patients, the change should be over 10.9, 7.8, and 5.1 for ACL-RSI, IKDC, and Lysholm scores, respectively. Likewise, when monitoring changes in a group of patients the values should be over 6.8, 7.8, and 4.8 for ACL-RSI, IKDC, and Lysholm scores, respectively.

The results of ACL-RSI in this study are comparable with those of research in other-language versions, such as Dutch (MCID, 2.6; MDC_ind_, 15.3)^14^ and English (MCID, 13.4; MDC_ind_, 26.6).^19^ All showed MCID values smaller than MDC_ind_. The present Lysholm score results with a large SRM value of 1.5 are in line with those of English-language research with an SRM of 0.9.^1^ The present IKDC results differ somewhat from those found in other-language versions. The Swedish, German, and Japanese versions showed MCID smaller than MDC_ind_ (Table 5).^8,10,18^ It was found that MCID was larger than MDC at the group level and MDC_ind_, which supports the use of IKDC to monitor groups or individuals over time. The differences between this study and other-language versions could be related to the differences of time interval selection that allowed knee function and confidence changes to be detected. For example, this study used a 6-month postoperative interval (at 0-6 months), whereas time intervals of 2 months were used in the Dutch version of ACL-RSI,^14^ 6 months (at 6-12 months) in the English version of ACL-RSI,^19^ 4 months in the Swedish version of IKCD,^18^ and 3 months in the Japanese version of IKDC.^8^

The clinical implications of the present study are 2-fold. First, knowledge of responsiveness of the PROMs helps the clinician in the interpretation of a change in score when assessing the status of patients after ACL injury over time using a PROM during treatment and rehabilitation. Second, to detect clinically important changes over time on group level, all 3 PROMs - ACL-RSI, IKDC, and Lysholm - could be used appropriately. However, at the individual level, use of IKDC seems the most appropriate.

**Table 5:** Responsiveness of Different Versions of the ACL-RSI, IKDC, and Lysholm Scores for Patients With ACL Injury*^a^*

Study	Language	MCID	SRM	MDC_ind_	MDC_gr_	SEM	Postoperative Time Interval
ACL-RSI							
Webster (2021)^19^	English	13.4	0.7	26.6	1.3	9.6	6 mo (at 6-12 mo)
Slagers (2019)^14^	Dutch	2.6	0.3	15.3	1.5	5.5	2 mo (at 3-9 mo)
Current study	Indonesian	6.8	1.6	10.9	1.1	3.9	6 mo (at 0-6 mo)
IKDC							
Tigerstrand (2017)^18^	Swedish	13.9	1.9	15.8	-	5.7	4 mo (at 0-4 mo)
Kummel (2018)^10^	German	-1.3	0.8	12.3	-	4.4	6 mo (at 0-6 mo)
Huang (2021)^8^	Japanese	10.7	-	14.6	-	5.2	3 mo (at 0-3 mo)
Current study	Indonesian	7.8	1.72	5.8	0.7	2.1	6 mo (0-6 mo)
Lysholm							
Briggs (2009)^1^	English	-	0.9	8.9	-	3.2	6 mo (at 0-6 mo)
Current study	Indonesian	4.8	1.5	5.1	0.6	1.8	6 mo (at 0-6 mo)

*^a^* Dashes indicate areas not reported. ACL, anterior cruciate ligament; ACL-RSI, Anterior Cruciate Ligament-Return to Sport after Injury scale; IKDC, International Knee Documentation Committee subjective knee form; MCID, minimal clinically important difference; MDC_gr_, minimal detectable change at the group level; MDC_ind_, minimal detectable change at the individual level; SEM, standard error of measurement; SRM, standardized response mean.

### Limitations

This study has some limitations. MCID was determined only by the predictive value method. MCID was not measured with other methods, such as receiver operating characteristic analysis, as others have done.^14^ However, this study used the logistic regression calculation by predictive value method as proposed by Terluin et al,^17^ which is an acceptable method for measuring responsiveness of the health scales. In addition, this study was conducted using the Indonesian version of those scales in an Indonesian population with ACL injury. These findings are not transferable either to the other-language versions or to other populations. Responsiveness of those scales should be determined in future research when using them in other particular populations.

## Conclusion

The Indonesian ACL-RSI, IKDC, and Lysholm scores indicated good responsiveness and can be used in the follow-up of patients after ACL injury, especially at the group level. In individual patients, IKDC seems more efficient than the other 2 scales in detecting clinically important changes over time after ACL treatment.
